# Iron-mineral accretion from acid mine drainage and its application in passive treatment[Fn FN0001]

**DOI:** 10.1080/09593330.2015.1118558

**Published:** 2016-01-23

**Authors:** K. Florence, D.J. Sapsford, D.B. Johnson, C.M. Kay, C. Wolkersdorfer

**Affiliations:** ^a^ Cardiff School of Engineering, Cardiff University, Cardiff, UK; ^b^ School of Biological Sciences, Bangor University, Bangor, Gwynedd , UK; ^c^ Department of Environmental, Water and Earth Sciences, Tshwane University of Technology (TUT), Pretoria, South Africa; ^d^ Laboratory of Green Chemistry, Lappeenranta University of Technology (LUT), Mikkeli, Finland

**Keywords:** Nanoparticles, aggregation, Fe oxidation, mine water remediation, schwertmannite

## Abstract

This study demonstrates substantial removal of iron (Fe) from acid mine drainage (pH ≈3) in a passive vertical flow reactor (VFR) with an equivalent footprint of 154 m^2^ per L/s mine water and residence times of >23 h. Average Fe removal rate was 67% with a high of 85% over the 10-month trial. The fraction of Fe passing a 0.22 µm filter (referred to here as Fe-filt) was seen to be removed in the VFR even when Fe(II) was absent, indicating that the contribution of microbial Fe(II) oxidation and precipitation was not the dominant removal mechanism in the VFR. Removal rates of Fe-filt in the VFR were up to 70% in residence times as low as 8 h compared with laboratory experiments where much smaller changes in Fe-filt were observed over 60 h. Centrifugation indicated that 80–90% of the influent Fe had particle sizes <35 nm. Together with analyses and geochemical modelling, this suggests that the Fe-filt fraction exists as either truly aqueous (but oversaturated) Fe(III) or nanoparticulate Fe(III) and that this metastability persists. When the water was contacted with VFR sludge, the Fe-filt fraction was destabilized, leading to an appreciably higher removal of this fraction. Heterogeneous precipitation and/or aggregation of nanoparticulate Fe(III) precipitates are considered predominant removal mechanisms. Microbial analyses of the mine water revealed the abundance of extracellular polymeric substance-generating Fe-oxidizing bacterium ‘*Ferrovum myxofaciens*’, which may aid the removal of iron and explain the unusual appearance and physical properties of the sludge.

## Introduction

Iron (Fe) (along with acidity and aluminium) is often the principal contaminant of concern present in acid mine drainage (AMD) from coal mines. Typically, this metal is also present with other potentially contaminating metals and metalloids such as As, Cu, Zn, Pb and Mn in AMD from metal mines.[1,2] It is advantageous to remove Fe in both settings and to remove it separately from other contaminants. One reason is the prevention of clogging/armouring of subsequent treatment cells which are used for the removal of acidity. For example, limestone is commonly used in passive treatment units such as open or anoxic limestone drains and Rapid and Alkalinity Producing Systems (RAPS) systems, and many other alternative reagents have also been tested (see, for example [3]). The longevity of these treatments can be compromised by clogging due to Fe (and Al) precipitates formed during AMD neutralization.[4] Compost bioreactors are often used to remove metals from mine drainage [5–7] and can be similarly compromised. Thus, it is advantageous if Fe can be removed in a ‘pretreatment’ (see, for example,[8]) stage so as to prolong the life of subsequent passive neutralization treatment steps which neutralize acidity and/or remove other contaminants. Furthermore, when Fe is removed separately with minimal other contaminants, the Fe may be more amenable to recovery and reuse. Potential applications are brick and cement manufacture [9]; phosphate removal in sewage treatment works [10–13] and pigment production.[14–16] Separating the iron, which often makes up the largest amount of the contaminant load, from other potential constituents such as other metals and organic debris can be advantageous in providing a less hazardous waste when landfill is intended.

There is a paucity of treatment systems specifically designed to target Fe removal at low pH. A few notable exceptions were the open pit lignite mine pilot-scale operation in Nochten, Germany,[17,18] and AMD treatment systems with Fe removal stages in the form of terraces, cascades or natural Fe oxidizing lagoons that are being incorporated into multi-stage treatment systems.[8,19] In these studies, the removal mechanism in the treatment is microbial Fe(II) oxidation and subsequent precipitation of Fe(III) minerals, with the particular Fe(III) mineral precipitated and the residual aqueous Fe concentration being dependent upon the pH [20–25] and related publications detail a vertical flow reactor (VFR) system which operates under aerobic conditions to remove Fe from circum-neutral pH coal mine water. This was based on earlier observations in the literature (see, for example,[26]) as well as observations from the field concerning the ubiquity of Fe(III) precipitates forming on the upper surface of vertical flow RAPS. On its course through the VFR system, mine water passes down through an unreactive gravel bed support media to aid in the accretion of Fe-bearing solids. The vertical flow design reduces the footprint compared with more traditional systems that require large areas of land.[25] Iron removal in the earlier VFR systems occurred by filtration of iron hydroxide particles that formed in the water column, heterogeneous oxidation of Fe(II) and precipitation of Fe(III) minerals,[25] cf.[26,27] This paper investigates the performance of a VFR for the removal of Fe at low pH and explores its removal mechanisms.

## Materials and methods

### Study site

It is estimated that there are tens of thousands of abandoned mining facilities including onshore mining and quarrying sites in the UK.[28] National Resources Wales (formerly known as the Environment Agency Wales) published ‘The Metal Mines Strategy for Wales’ [29] which lists the top 50 most polluting sites in Wales that were identified following monitoring 5042 km of river stretches, 108 km of which failed ecosystem objectives as a direct consequence of pollution from abandoned mines.[30] Thirty-eight of those top 50 sites are located in the Mid-Wales district of Ceredigion. Cwm Rheidol (a former Pb/Zn mine) is listed in the Metal Mines Strategy for Wales as one of these top 50 polluting mines.[29] The primary sulphide minerals at Cwm Rheidol were sphalerite, galena, arsenopyrite, some pyrite and marcasite.[31] Despite having been closed for almost a century, the mine continues to produce AMD, which flows untreated into the Afon Rheidol.[32] AMD discharges from two adits that extend up to 500 m into the hillside, where they form a junction with the Ytsumen mine complex.[33] The VFR trial was set up to take a proportion of the flow that comes from the lower number 9 adit. Typically, the mine water has a of pH 2.9, and has concentrations of Fe, Zn, Al, Cd and Pb < 90 mg/L, 125 mg/L, 25 mg/L, 0.1 mg/L, 0.06 mg/L respectively, and a sulphate concentration of 1500–2000 mg/L.

### VFR construction

**Figure 1.**
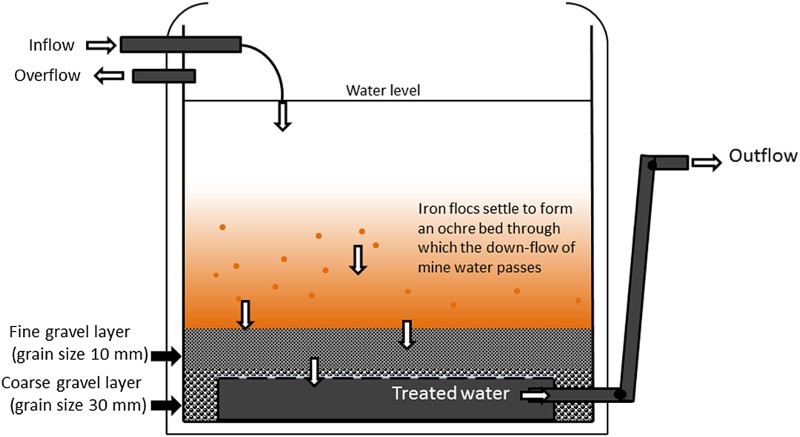
Conceptual diagram of the VFR field reactor (not to scale).

The gravity fed VFR system was adapted from the original design used in an initial field trial at the Taff Merthyr site in South Wales,[23,34] using an adapted 1 m³ intermediate bulk container. A length of coiled, slotted drainage pipe was placed in the bottom of the tank. 30 mm angular coarse grained siliceous chips were added to the container to secure the pipe and provide a stable base on top of which a 200 mm depth layer of 5–10 mm grain size siliceous gravel was added. The tank was initially filled through a perforated medium to dissipate the flow allowing a build-up of ochre from the flow through of mine water. As the ochre built up and the permeability of the bed decreased, a ball and valve tap were fitted, which was adjusted to prevent the system from overflowing (Figure 1). A down flow of mine water was then maintained through the water column and through the VFR bed. Hydraulic head was set initially to 0.36 m and at later stages to 0.61 m via an adjustable swan neck through which the treated water was discharged.

### Field monitoring

Geochemical parameters and flow rates were measured and recorded over the trial period of 10 months. Filtered (0.22 µm) and unfiltered water samples were collected from the inflow and outflow for metals concentrations and samples were acidified with 20% (v/v) HNO_3_ and stored at 4°C prior to analysis. In addition, filtered, non-acidified samples were taken for anion analyses. For clarity, throughout this paper, samples that were filtered through a 0.22 µm filter are reported as ‘Fe-filt’, unfiltered and acidified samples referred to as ‘Fe-tot’. All aqueous elemental analyses in this study (unless otherwise specified) were carried out using a Perkin Elmer Optima 2100DV ICP-OES. The instrument was calibrated using three calibration standards (0.10, 1.00 and 10.00 mg/L) made up in deionized water from certified analytical standards. Anion analysis was performed using a Dionex ICS-2000 Ion Chromatograph with an AS11-HC column using three calibration standards (0.10, 1.00 and 10.00 mg/L) made up in deionized water from certified analytical standards. On-site spectrophotometric determinations of Fe(II) concentrations were undertaken on 11 sampling occasions using filtered samples and a portable Merck NOVA60 spectrophotometer with Merck Fe test cells. Laboratory determinations of Fe(II) were carried out using a Hitachi U1900 spectrophotometer on samples that were filtered and preserved with 20% (v/v) HCl. Both the field and laboratory spectrophotometric analysis for Fe(II) used 2,2′ bipyridine as the complexing agent. Where data for Fe-Filt and Fe(II) analyses were available together, this allowed calculation of Fe(III) by difference. All field measurements of pH, electrical conductivity (EC), redox-potential (ORP) and temperature were taken using Hanna combination meters HI-9828. Dissolved oxygen (DO with Clarke-sensor), EC and pH probes were calibrated at each site visit. Flow rates were measured, using a bucket and stop watch, the procedure repeated three times and an average of the three measurements taken as the final value. A falling head permeability test was conducted on the VFR following the method of [35] during the final drain down of the system at the end of the trial period.

### Microbial characterization of mine water

DNA was extracted from 0.2 µm (pre size) membranes through which approximately 1–1.5 L of Cwm Rheidol mine water had been filtered. The membranes were cut into small pieces and DNA extracted using a MO-BIO Ultraclean Soil DNA Isolation kit (MO-BIO, USA) following the manufacturer's instructions. Bacterial and archaeal 16S rRNA genes were amplified from the extracted DNA using GoTaq Hotstart PCR (Promega) and either the bacteria-specific primers 27F (5′- AGAGTTTGATCMTGGCTCAG-3′) and 1387R (5′-GGGCGGWGTGTACAAGGC-3′), or the archaea-specific primers 20F (5′-TCCGGTTGATCCYGCCRG-3′) and 915R (5′- GTGCTCCCCCGCCAATTCCT-3′). The conditions used for polymerase chain reaction (PCR) amplification of the bacterial genes were: 95°C–5 min, 30 cycles of 95°C–30 s, 55°C–30 s, 72°C–1.5 min, and a final extension of 72°C–10 min. PCR amplification of the archaeal genes were: 95°C–5 min, 30 cycles of 95°C–30 s, 62°C–30 s, 72°C–1.0 min, and a final extension of 72°C–10 min. The 27F/20F primers used were labelled with the Cy5 fluorophore to allow assessment of the microbial communities using terminal restriction enzyme fragment length polymorphism (T-RFLP) analysis.[36] Amplification of 16S rRNA genes was performed in triplicate to prevent PCR bias effects, and the combined reactions were purified to concentrate the DNA and to remove buffer salts and excess reagents. The PCR products were then separately digested (in 10 µL aliquots) using 5 units of one of the three restriction endonuclease enzymes (HaeIII, CfoI and AluI). Digested DNA (1 µL) was loaded onto a 96-well plate containing sample loading solution (formamide) and DNA nucleotide standards (600 bp) and analysed by capillary electrophoresis using a CEQ8000 (Beckman Coulter, UK). The terminal restriction fragments obtained were with those in a database maintained at Bangor University to facilitate the identification of indigenous bacteria and archaea in the mine water samples.

### Colloid size determination

A centrifugation method adapted from the method presented in [37] was used to determine the size distribution of particulates in the mine water. Twenty tubes of fresh mine water were collected in the field (10 × 2 to duplicate the experiment). Parallel centrifugation using a SIGMA^®^ 6K15 centrifuge of each 2 × 50 mL of raw mine water was carried out at rotor speeds of 300, 500, 700, 1000, 3000 as well as 5000 rpm over a period of 1 hr and 3500 rpm for 2 h due to the limitation of the centrifuge instrument speed. Equivalent relative centrifugal force (RCF) values (relative centrifugal force = *g*-force) at a given point in the centrifuge tube were calculated according to the following equation:(1) 
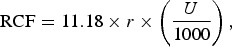
with *r* being the distance between the rotation axis and the particle in the centrifuge tube in cm, measured with a ruler and *U* rotations per minute (rpm). After centrifuging, the supernatant was syringed from the centrifuge tube using a pipette and the relative water level measured with a ruler. The sample was transferred to a new sample container, acidified with 20% (v/v) HNO_3_ and the total unfiltered concentrations determined by ICP-OES. Assuming that the particles still present are spherical, their maximum size in the supernatant was then calculated using the below equation:(2) 
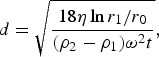

*d* being the diameter of particle, cm; *ρ*_1_ the density of water at 25°C, 0.997 g cm^−3^; *ρ*_2_ the density, g cm^−3^; *ρ*_2_ 3.96 g cm^−3^ for the iron mineral; *r*_0_ the distance of water level in vial to rotation axis before taking sample, cm; *r*_1_ the distance of water level in vial to rotation axis after taking sample, cm; *t* the duration of centrifuging, s; *η* the viscosity of water at 25°C, 0.008941 g cm^−1^ s; *ω* the angular velocity, 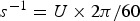


### Iron removal experiments

Two experiments were carried out to investigate the effect of stirring, aeration and solids addition on the rates at which Fe-filt was removed from solution. In both cases, untreated mine water was collected from the Cwm Rheidol lower adit discharge pipe and the concentration of Fe(II) measured spectrophotometrically using the 2,2′ bipyridine method. Mine water was returned to the laboratory for experiments. pH, ORP (mV), EC (µS/cm) and temperature (°C) were recorded and the Fe-filt was measured by ICP-OES. Measurements and samples were collected at the start of the experiment and after 50 h. In the first experiment, two reactors were used: one aerated/agitated using a small submersible pump while the second reactor was left still (static and undisturbed). In the second experiment, where there was no Fe(II) observed in the sample initially, four reactors were used each with 500 mL of freshly collected mine water, reactor (a) static, (b) stirred, (c) aerated, (d) stirred + known mass of VFR sludge. About 0.5 g of wet sludge collected from the VFR was added to reactor (d); later drying of a subsample of the sludge indicated that the dose of VFR sludge added was 0.72 g/L.

### Physicochemical characterization of the VFR sludge

The settling velocity of an aliquot of freshly collected sludge from the VFR was determined by measuring the distance at which the particles at 2.5% w/v concentration settled over time in a measuring cylinder. After completion of the field trial, the water was drained and the sludge collected for chemical and mineralogical characterization. Elemental determination was done by 4-acid microwave acid digestion and inductively coupled plasma - optical emission spectroscopy (ICP-OES) analysis. Total sulphur was determined using a Leco S Furnace. Mineralogical characterization was carried out by X-Ray Diffraction (XRD) using a Philips PW3830 X-Ray generator with a Cu anode (K 1.54 Å), Philips PW1710 diffractometer controller and ×Pert High Score plus software. Environmental Scanning Electron Microscope (ESEM) was used to examine the mineralogical structure of the precipitates. Samples were mounted on self-adhesive 12.5 mm pin stubs, carbon coated and analysed using a FEI XL 30 FG ESEM fitted with a Peltier-cooled specimen stage using an Oxford Instruments INCA ENERGY X-ray analyser.

To characterize the iron pools, five stages from the sequential extraction technique of Poulton and Canfield [38] were used (Table 1). Six dried samples were crushed to <63 μm with an agate mortar prior to extraction and analysis (one sample for each of the five stages plus one for Fe_total_). An aliquot of 150 mg from each sample was utilized for the iron extraction. The extractions were performed in 50 mL polypropylene centrifuge tubes using 10 mL of extractant solutions, except for the boiling HCl stage in which 5 mL of solution and a glass tube was used. The solutions were finally filtered with 0.2 μm Nalgene syringe filters and analysed for iron and trace elements content by ICP-OES.

**Table 1.** Details of the sequential extraction scheme performed on the VFR ochre from Poulton and Canfield [34].

Extraction	Terminology	Target phases
Na Acetate pH 4.5, 24 h	Fe_carb_	Carbonate Fe, including siderite and ankerite
Hydroxylamine – HCl, 48 h	Fe_ox1_	Ferrihydrite, lepidocrocite
Dithionite, 2 h	Fe_ox2_	Goethite, akaganéite, hematite
Oxalate, 6 h	Fe_mag_	Magnetite
Boiling 12 N HCl, 5 min	Fe_PRS_	Poorly reactive sheet silicates Fe

## Results and discussion

### Field trial results

Table 2 shows the influent and effluent chemistry of the mine water during the VFR trial. An average of 67% of the iron present was removed by the VFR system during the 10-month trial period during which the iron oxyhydrate and sulphate accreted on top of the gravel bed (i.e. did not penetrate the gravel). Flow rates varied between 0.12 and 1.1 L/min, with a mean recorded flow of 0.38 L/min through the 1 m² bed. The VFR maintained this flow with a 0.61 m mean hydraulic head. The nominal retention time (volume/flow rate) reported in Table 2 should be treated as an upper estimate, as the hydraulics are unlikely to be ideal plug-flow. The flow treated equates to an area of 158 m² of bed area per litre per second of mine water flow, which is not unreasonable in comparison with other sizings for passive systems given that Fe is being removed directly from the AMD without reagent addition. For example one of the criteria used for the design of is 100 m^2^ L^−1^ s^−1^ of mine water flow [39] for net-alkaline ferruginous mine water which is considered relatively easy to treat compared with AMD. A more comparable mine water treatment system [8] demonstrated up to 38% removal of iron from low pH mine water with water flowing over Fe ‘stromatolites’ and on into a lagoon with a footprint of 67 m^2^ L^−1^ s^−1^ of mine water flow.

**Table 2.** VFR field parameter measurements.

Date	Flow rate (L/min)	pH	pH	D.O (mg/L)	D.O (mg/L)	*E*_H_ (mV)	*E*_H_ (mV)	Temp (°C)	Temp (°C)	Fe-Tot (mg/L)	Fe-Tot (mg/L)	Fe-Tot Removal %	Fe-Filt (mg/L)	Fe-Filt (mg/L)	Fe (Filt) Removal %	Fe(II) (mg/L)	Fe (II) (mg/L)	ΔFe-Filt (mg L)	RT (h)
	*E*	*I*	*E*	*I*	*E*	*I*	*E*	*I*	*E*	*I*	*E*		*I*	*E*		*I*	*E*	*I*/*E*	
21 June 2011	0.71	2.98	3.01	8.47	7.33	684	716	13.54	14.14	126	60.81	51.74	126.59	65.3	48.42	na	na	61.29	16
11 July 2011	0.46	3.29	3.38	7.54	7.81	670	742	15.41	18.01	23.25	17.1	26.45	16.45	15.8	3.95	na	na	0.65	24
20 July 2011	0.38	3.33	3.26	9.51	4.12	670	764	14.37	16.04	119.6	41	65.72	116.2	41.16	64.58	na	na	75.04	31
3 August 2011	0.25	3.03	3.09	5.83	4.38	678	763	16.97	16.26	104.9	31.37	70.10	64.87	31.82	50.95	37.1	<0.2	33.05	47
11 August 2011	0.11	2.58	3.05	6.49	5.89	758	776	17.88	19.64	80.56	32.09	60.17	64.87	31.82	50.95	0.58	<0.2	33.05	85
22 August 2011	0.3	3.42	3.58	6.9	4.30	673	710	15.27	14.48	146.2	53.34	63.52	138.7	52.45	62.18	na	na	86.25	48
30 August 2011	0.2	3.03	3.02	8.35	6.66	673	762	12.02	12.64	155.1	53.9	65.25	135.5	51.18	62.23	10.2	na	84.32	59
19 September 2011	0.2	3.05	3.00	5.66	5.55	767	678	12.88	12.19	87.54	23.63	73.01	82.5	23.86	71.08	12.3	<0.2	58.64	70
25 September 2011	0.29	3.04	3.06	8.85	5.40	663	757	12.28	14.05	101	20.75	79.46	77.17	20.19	73.84	na	na	56.98	30
26 September 2011	0.24	2.83	2.63	7.66	4.08	668	728	16.01	19.67	84.91	18.13	78.65	72.2	17.76	75.40	na	na	54.44	58
4 October 2011	0.26	2.5	2.41	9.20	6.31	664	718	12.40	12.26	97.09	28.19	70.97	91.31	27.78	69.58	32	<0.2	63.53	60
7 October 2011	0.24	2.65	2.68	7.19	6.73	741	724	15.08	15.38	110.9	28.91	73.93	102.5	31.23	69.53	35	<0.2	71.27	52
21 October 2011	0.31	3.01	3.15	7.70	10.02	638	648	10.44	11.46	84.13	23.64	71.90	75.53	23.92	68.33	33.1	1	51.61	40
4 November 2011	0.19	2.88	2.59	9.32	6.10	662	727	10.52	9.47	37.82	24.35	35.62	16.68	3.97	76.20	4.44	<0.2	12.71	50
18 November 2011	0.2	2.77	2.63	9.16	7.75	359	722	11.14	10.96	110.4	29.45	73.33	103.93	29.05	72.05	43.5	1.18	74.88	72
2 January 2012	0.1	2.45	2.37	9.13	6.83	718	726	6.75	6.74	95.67	14.56	84.78	35.83	11.53	67.82	4	<0.2	24.3	112
18 March 2012	0.23	2.38	2.32	7.07	6.45	773	741	9.18	10.81	79.15	23.51	70.30	72.32	24.74	65.79	na	na	47.58	38
7 June 2012	1.1	3.08	2.54	8.30	7.20	476	448	13.04	14.09	102.1	24.28	76.22	91.17	24.49	73.14	na	na	66.68	6
30 June 2012	1.1	4.86	4.94	6.60	5.54	505	492	14.37	13.63	75.3	20.37	72.95	69.26	21.6	68.81	27.6	4.6	47.66	10
4 July 2012	0.75	2.87	2.92	7.42	5.89	485	516	13.40	12.51	79.2	17.31	78.14	63.69	17.1	73.15	17.2	<0.2	46.59	15
Mean	0.38	2.83	2.77	7.8175	6.217	646	693	13.15	13.72	95.04	29.33	67.11	80.86	28.33	63.4	21.42	0.69	52.53	46

Note: I, influent; E, effluent; na: not applicable or not measured; the mean of the pH was calculated by using H^+^ and converting it back to pH.

The influent pH of the mine water was consistently low at pH 2.4–4.9. No statistically significant difference between the influent and effluent pH was seen, indicating that hydrolysis of ferric iron (a proton producing reaction) is not likely to be an important reaction in the VFR. The VFR did have a measurable oxygen demand with a slight decrease in DO across the VFR in the majority of cases; the mean effluent DO was 6.2 mg/L compared with the mean influent DO of 7.8 mg/L (on average a 1.6 mg/L decrease, which is statistically significant at *α* = 0.05 and *σ* < 0.0001). ORP data (corrected to the standard hydrogen electrode, i.e. *E*_H_ values) indicate that for the majority of the sampling occasions, there was a slight increase in *E*_H_ between the influent and effluent. The observed temperature changes were small and correlated with seasonal air temperature.

Where Fe occurred in the influent mine water as particulate (> 0.22 µm) Fe(III) precipitates (difference between Fe-tot and Fe-filt in Table 2), the removal mechanism was attributed to filtration of these particles (see, for example, [25]). However, in the influent on average approximately 85% of the Fe was in the Fe-filt fraction (i.e. passing a 0.22 µm filter) and in the effluent on average 99% of the Fe was in the Fe-filt fraction. Interestingly, an average 53 mg/L of Fe is removed from the mine water and this amount is consistently higher than can be explained by filtration of particulate Fe(III) > 0.22 µm only. Thus, on its passage through the VFR, the Fe-filt fraction is removed by some other mechanism(s). Possible candidate mechanisms depend on the Fe-speciation within the Fe-filt fraction and include: (i) microbial Fe(II) oxidation and precipitation of Fe(III) solid phases – on the occasions where Fe(II) was present; (ii) filtration of nanoparticulate-Fe(III)_(s)_ which are < 0.22 µm (and therefore not retained when samples were filtered); (iii) heterogeneous precipitation of Fe(III) and (iv) adsorption of dissolved Fe(II) and Fe(III) to existing precipitates. This latter explanation is highly unlikely because there were not sufficient particulates entering the system to sustain adsorption as a long-term removal mechanism.

When analyses were carried out on site it was apparent that ferrous iron accounted for only a small proportion of the total influent Fe (highest observed was 42% of the influent Fe-filt on 18 November 2011). When present in the influent ferrous iron was effectively oxidized in the VFR, with 7 of the 11 sampling occasions falling below the detection limit of the analytical method used (0.2 mg/L). The smallest removal of ferrous iron was 83% on 30 June 2012 (with 10 h, having had one of the shortest residence times). It was concluded that although concentrations were variable, Fe(II) was typically a small component of the Fe-filt fraction and that it was removed effectively in the VFR. Therefore (and of great significance in relation to other reports on low pH Fe removal), Fe(II) oxidation and precipitation of Fe(III) minerals cannot alone explain differences in Fe-filt between influent and effluent.

**Figure 2.**
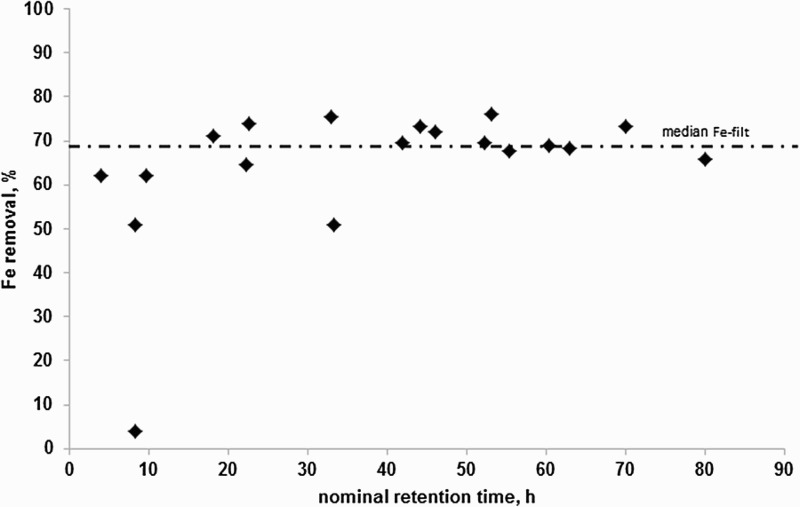
Removal (%) of Fe-filt (see text) versus nominal retention time from the Cwm Rheidol field trial.

 Figure 2 gives the result of the Fe-filt removal versus nominal residence time. A clear trend was observed and it shows that the amount of Fe-filt removed from the mine water reached a maximum of approximately 70% and that this was independent of residence time for times of >23 h. This demonstrates that there was a proportion of the influent Fe-Filt fraction that could be removed by the VFR and a proportion of the influent Fe-Filt fraction that passes the treatment system regardless of residence time; these fractions were in an approximate ratio of ≈70:30. Given that Fe(II) is not prevalent in the water the identity of the Fe-Filt fraction is Fe(III) and the removal mechanism is by either mechanism (ii) or (iii) (see above), the lack of observable decrease in pH across the VFR suggests (ii) is unlikely. The residual Fe concentration passing through the system is thought to be dissolved Fe(III) remaining in solution in accordance with the solubility limit of mineral phases precipitated at the operating pH of the system.

### Hydraulic conductivity

As can be seen from the consistent flow rate data (Table 2), the permeability of the VFR remained more or less consistent after the initial filling phase. The mean effective hydraulic conductivity of the bed of precipitates in the VFR, measured at the end of the field trial was 2.08 × 10^−5^ m/s. This is classified as ‘pervious’.[40] Results compare well with those reported by Barnes,[41] who determined an average hydraulic conductivity of 3.1 × 10^−5^ m/s in a VFR treating net-alkaline coal mine drainage. Consequently, the results for the VFR at Cwm Rheidol and Taff Merthyr are in the same range despite differences in mine water chemistry and operation time (379 and 287 days, respectively).

### Mine water microbiology

T-RFLP analysis of the 16S rRNA genes amplified from Cwm Rheidol mine water showed a relatively simple bacterial community dominated (approximately 50% relative abundance) by the iron-oxidising β-proteobacterium ‘*Ferrovum myxofaciens’.*[42] Other iron-oxidizers identified were the psychrotolerant iron/sulphur-oxidizing acidophile *Acidithiobacillus ferrivorans* (10% relative abundance), *Leptospirillum* sp. (most likely *L. ferrooxidans*, given the water temperature; 9% relative abundance) and the mesophilic iron/sulphur-oxidizing acidophile *Acidithiobacillus ferrooxidans* 5% relative abundance, respectively). ‘*Fv. myxofaciens*’ has widespread distribution in acidic (pH 2–5) mine waters throughout the world [43] and its occurrence (and apparent dominance) in Cwm Rheidol AMD was therefore not unexpected. It is a specialist bacterium, known only to oxidize ferrous iron to ferric. It has been used to oxidize iron in synthetic mine water in laboratory-scale bioreactors,[44] where its propensity to generate extracellular polymeric substances (EPS), allowing it to attach to surfaces and to grow as stable macroscopic ‘streamers’ in flowing AMD were found to give it a substantial advantage over other iron-oxidizing acidophiles. Massive accumulations of acid streamer growths in mine waters elsewhere in Wales have been found to be dominated by ‘*Fv. myxofaciens*’ and *At. ferrivorans*.

### Sludge settling velocity

**Figure 3.**
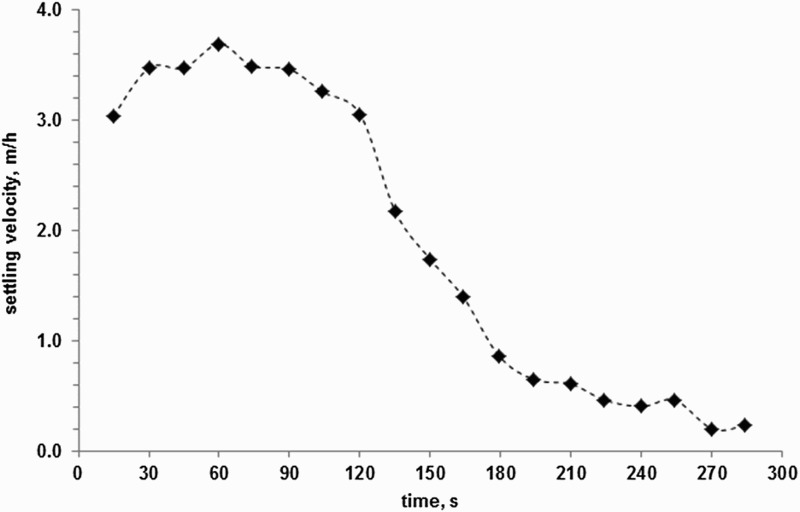
Settling velocity versus time for the freshly sampled VFR sludge.

**Figure 4.**
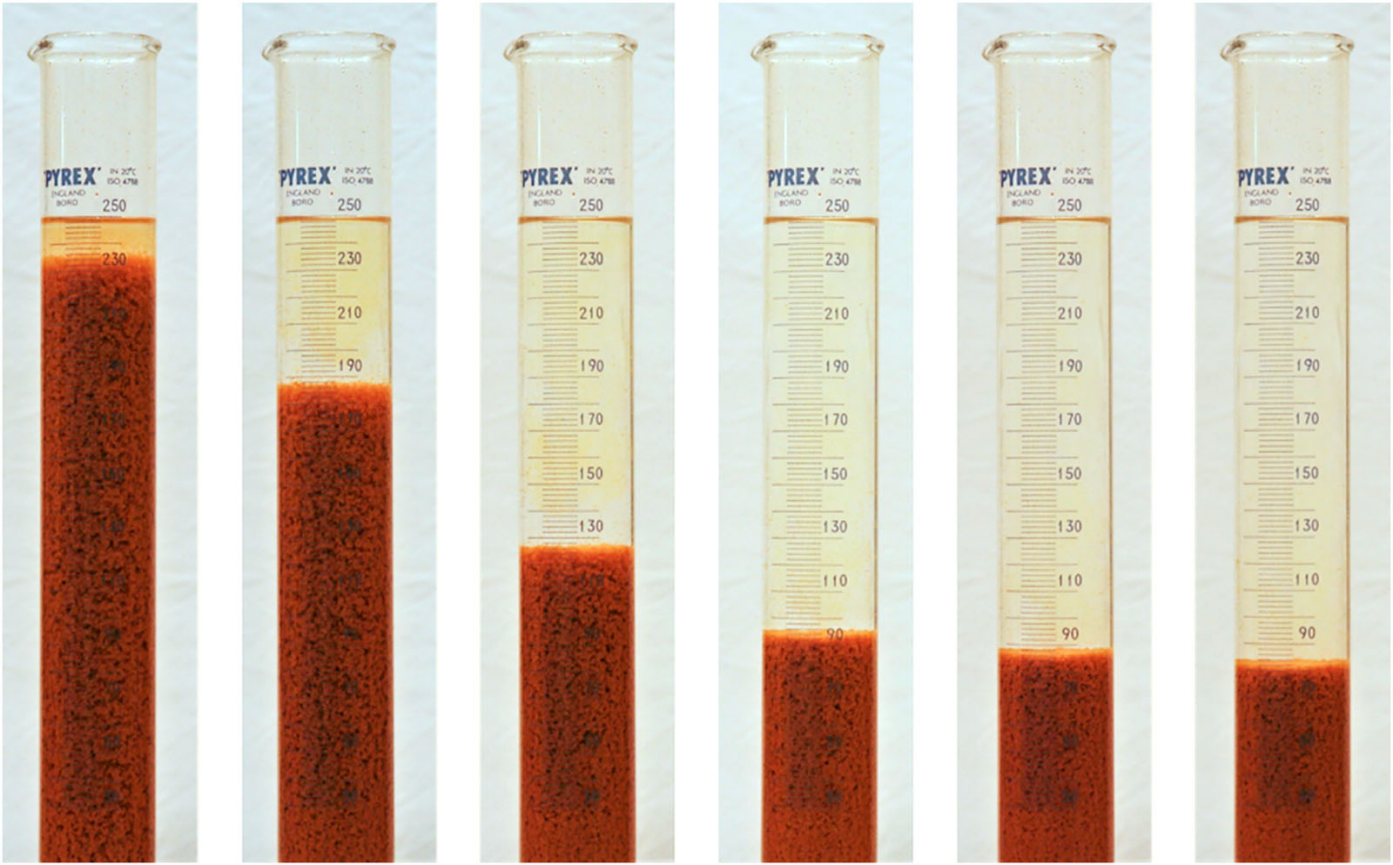
Time sequence images of the settling VFR precipitates, 15, 60, 120, 194, 240, 284 s. Note clarity of supernatant.

A mean settling velocity of 3.4 m/h was recorded within the first 120 s of the sludge settling experiment. This initial rapid linear settling behaviour slowed thereafter, stabilizing at approximately 0.2 m/h. The initial settling rates of the VFR sludge were substantially faster than has been previously observed for passive treatment sludge (between 0.006 and 1.2 m/h at a solids concentration of 2% w/v [45]) but not as high as high as observed for high density sludge (HDS) where polymer flocculant is added. Example average settling velocities measured for sludge from an HDS plant were 17 m/h (average initial solids concentration of 2.7% w/v).[46] The VFR sludge demonstrated these settling rates without the addition of polymer flocculants that are usually added in active treatment systems (see, for example [47]). Interestingly, the VFR sludge had the appearance of polymer-dosed sludge (Figure 4) and in addition to the settling velocity, the clarity of the supernatant water is unusual for passive mine water treatment sludges that have been stirred and allowed to settle. The authors postulate that this appearance and unusually high settling rate are related to the presence and prevalence of ‘*Fv. myxofaciens’* whose EPS could act as a natural polymer flocculent in the sludge (Figure 3).

### Sludge analysis

Sludge analysis shows (Table 3) that the VFR sludge was predominantly an iron-mineral sludge and that only minor amounts of other metals/metalloids were associated with the sludge. This result is in line with the metal removal data from the field trial. Interestingly, the sludge Fe and S content is almost identical to the schwertmannite composition reported by another similar study.[8] The results of the sequential extraction show that Fe is bound to the following extractions steps: Fe_carb_ 2.7%, Fe_ox1_ 14%, Fe_ox2_ 82%, Fe_mag_ 1.5% and Fe_PRS_ 0.03%, demonstrating that the majority of the Fe minerals is in the diothionite reducible phase. Phases targeted by this extraction step Fe_ox2_ include goethite and akaganéite.[38]

**Table 3.** Weight % of constituents of the VFR sludge.

Constituent	Weight%
Fe	36.11
S	4.40
Cu	0.10
Al	0.08
Ca	0.06
K	0.06
Zn	0.04
As	0.01

**Figure 5.**
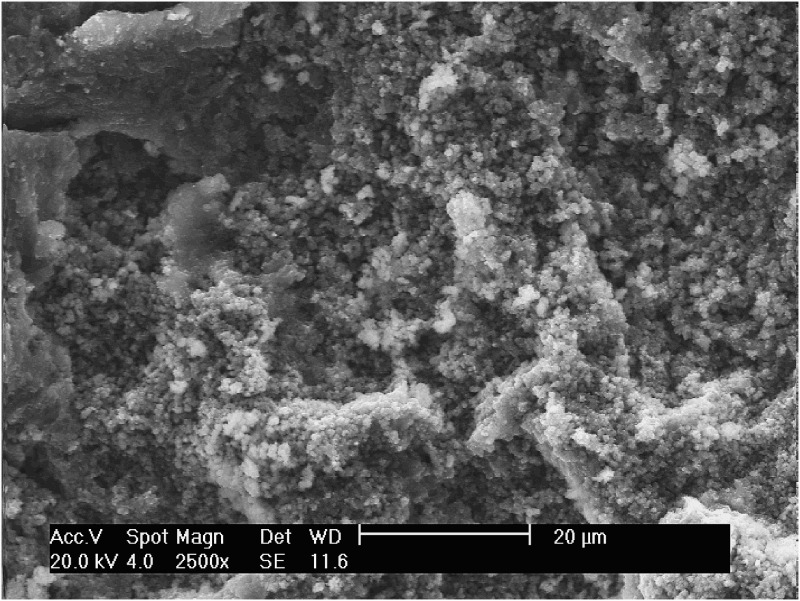
SEM image showing the morphology of the Cwm Rheidol Fe precipitates collected from the operating VFR.

Iron precipitates from AMD environments are described as being a variety of poorly ordered oxides and hydroxysulphates.[48–50] The most common Fe mineral that forms by direct precipitation from pH 2.8 to 4 water and sulphate concentrations between 1000 and 3000 mg/L is schwertmannite (see, for example,[44]) with an optimum pH range for precipitation between 2.8 and 3.2.[20] XRD analysis of the Cwm Rheidol VFR sludge displayed a generally amorphous signature but with some characteristic peaks of schwertmannite and goethite cf.[51,52] The ESEM image for the sample taken from the VFR during operation (Figure 5) shows precipitates with an indistinct morphology. The sample's spectrum data gave an indicative composition of 41.1% O, 56.16% Fe and 3.74% S, comparable to the analytical results (Table 3). PHREEQC modelling [53] using the minteq.dat v4 database and a range of manually inputted schwertmannite solubility products from log *K*_sp_ of 7.06 [54] to 18.5 [20] indicates that the original mine water is oversaturated with respect to schwertmannite with calculated SI between 13.10 and 1.66 respectively.

### Fe removal mechanisms

**Figure 6.**
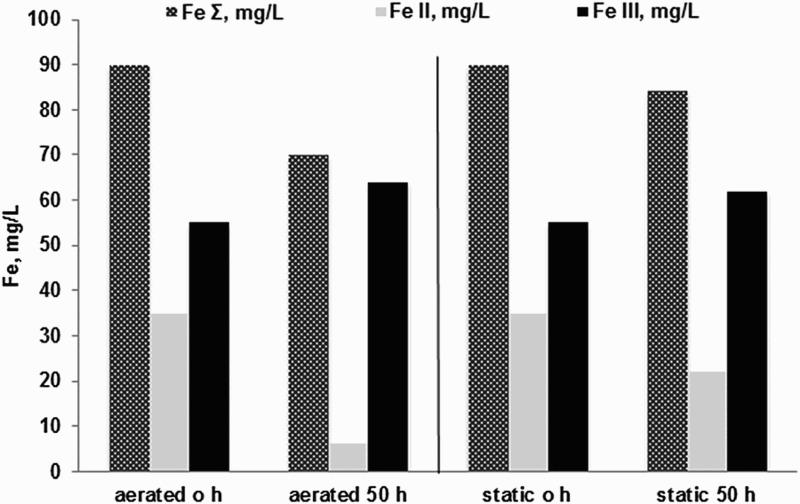
Fe-filt, Fe(II) and Fe(III) concentrations in aerated/agitated and static experiments of Cwm Rheidol inflow mine water after a 50 h reaction time.

Results from the initial laboratory experiment (Figure 6) demonstrate that total Fe decreased slightly in both aerated and static experiments but that a greater decrease was seen in the aerated experiment. In this case, there was substantial Fe(II) present in the mine water initially, and the decrease in Fe correlates with the decrease in Fe(II). The likely mechanism, consistent with the microbial analyses above and other publications, (see, for example,[55]) is microbial Fe(II) oxidation and precipitation of schwertmannite.

**Figure 7.**
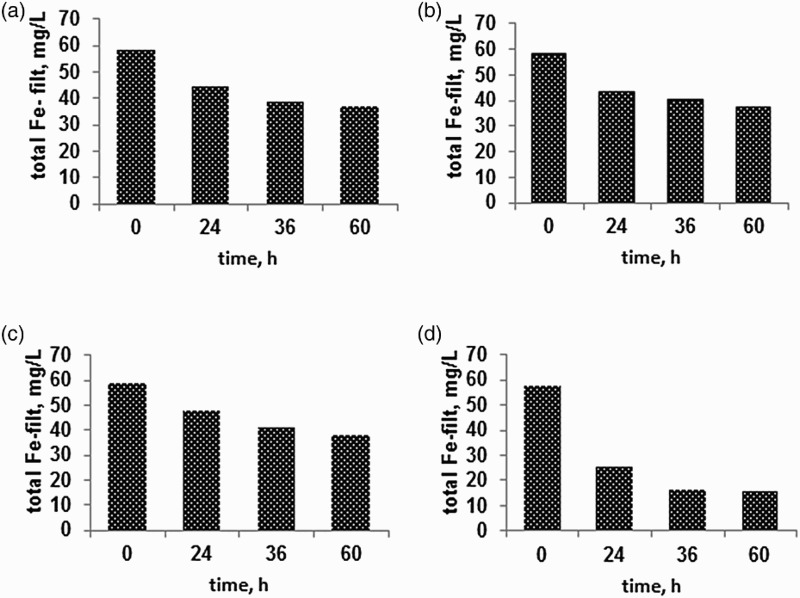
Fe-filt in (a) still reactor; (b) stirred reactor; (c) aerated reactor and (d) stirred with sludge added (0.72 g/L) reactor using Cwm Rheidol inflow mine water over a 60 h reaction time.

However, for the second experiment, over a duration of 60 h, Fe(II) was initially below limits of detection (Figure 7). The results for Fe-filt concentrations (Figure 7) over the 60 h in static, stirred, aerated and stirred with VFR sludge reactors showed that Fe-filt decreased in all cases from 58.9  to 37.1 mg/L, 58.4 to 33.7 mg/L, 58.8 to 37.9 mg/L, and 58.1 to 16.2 mg/L, respectively. This corresponds to a removal rate of approximately 35% of the Fe-tot concentration in the static (a), stirred (b) and aerated (c) reactors whereas 72% of the Fe was removed in the (d) stirred reactor with VFR solids added.

The length of the experiment is comparable to some of the longer residence times in the field VFR trial where typically 65–70% of the Fe-filt was removed (Figure 2). In the absence of ‘dissolved’ Fe(II), the Fe-filt fraction comprises either nanoparticulate or truly dissolved ‘molecular’ Fe(III) and the corresponding removal mechanisms are precipitation or aggregation/flocculation. Stirring made no obvious difference to the rate of Fe-filt removal which would be expected for an aggregating/flocculating system where particle sizes are <<1 µm and flocculation is perikinetic. The improved removal of Fe-filt after the addition of VFR sludge could indicate sorption of aqueous Fe(III) or more likely that presence of larger particles lead to improved capture/enmeshment of nanoparticulate iron precipitates. The latter mechanism might be expected to be enhanced by the presence of microbial polymers associated with the sludge as discussed above.

The centrifuge study was used to determine the size distribution for particulate Fe(III) in Cwm Rheidol mine water. Table 4 gives the results of the supernatant centrifuge analysis, with the equivalent stokes diameters of particulates from Equation (2) that would be removed at various centrifugation speeds. Data for Fe are shown and compared against data for Ca, Mg, Al and Zn. Ca and Mg are expected to be truly dissolved in this pH range and as expected, no decrease in the concentrations of Ca and Mg with increased centrifuge speed were observed, commensurating to these species being truly dissolved. Based on the experimental data, also Al and Zn seem to form no colloids above 35 nm at the pH of the Cwm Rheidol mine water. The Fe data show that Fe does decrease as centrifugation speed increases, however 87% of the iron measured in the raw mine water was still present in the mine water following centrifugation for 2 h at 3500 rpm which corresponds to a Stokes diameter of 35 nm. This indicates that the Fe present in the mine water sample was <35 nm in size.

**Table 4.** Results of the centrifuge experiment to determine particle size distribution of Fe compared to Ca, Mg, Al and Zn.

Sample #	Centrifuge speed (RPM)	Centrifuge time (h)	Particle diameter (nm)	Analyte concentration (mg/L)				
				Fe	Ca	Mg	Al	Zn
Raw mine water	–	–	–	99.6	59.5	39.7	27.7	81.6
0	–	–	–	98.2	59.2	39.3	27.84	82.8
1	300	1	683	96.6	63.3	41.4	29.3	84.9
2	500	1	417	92.8	62.1	40.2	28.4	83.7
3	700	1	293	82.1	62.3	40.6	29.2	85.0
4	1000	1	207	91.4	60.2	40.1	28.2	82.9
5	2000	1	101	89.0	61.3	40.2	28.5	84.1
6	4000	1	50	85.7	61.3	40.9	29.2	83.7
7	5000	1	39	84.5	72.7	41.4	28.9	87.1
8	3500	2	35	86.9	61.2	40.9	29.1	83.1

Whether or not the iron is or can be defined as nanoparticulate or truly dissolved is at this scale somewhat arbitrary and operationally defined.[56,57] There is no general consensus in the literature, under which threshold size particles can be considered ‘truly dissolved’, and the distinction between the two is analytically challenging.[58] Commonly, 1–1000 nm are referred to as colloids, 1–100 nm are nanoparticles and several authors call particle sizes under 30–50 nm ‘truly dissolved’.[59] Yet, 1 nm is still larger than the size of a molecule which is in the range of several Ångstroms but in the range of hydrated ions.[60] Nanoparticulate Fe has been noted in mine water before by Zänker et al,[61] who determined in a study of mine water from a former Zn–Pb–Ag mine colloid concentrations of >1 g/L with the prevalent particle size being <5 nm. The precise identity of the Fe-filt fraction may be important at determining the applicability of VFRs for Fe removal from other AMD sites.

To explain this behaviour of the size distribution, and what makes the Cwm Rheidol site unique, the whole Cwm Rheidol system must be taken into account. Before the mine water enters the VFR, it flows nearly 500 m through an aerated adit [31] and more than 100 m through the mine workings, where a substantial amount of Fe precipitates, as can be seen from visual inspection. Thereafter, the mine water is drained into a 200 m long drainage pipe, which also shows ochre precipitates. It is therefore very likely that most of the larger particles already sedimented during the course of the mine water through the mine workings, the adit and the drainage pipe. Consequently, the mine water entering the VFR can already be considered pre-treated and the particle size distribution is a result of size fractionation.

In terms of practical application of the VFR at the Cwm Rheidol site, it was shown during the field trial that a mean flow of 0.38 L/min was treated in the VFR using an area of 1 m^2^. Using the mean flow of 3 L/s from the number 9 adit, a 474 m² VFR (e.g. 19 m × 25 m) would be sufficient to remove an average of 68% of the Fe in the total flow from the number 9 adit for at least one year without maintenance. In terms of general design guidance, this equates to a footprint of 154 m^2^ per L/s of mine water flow and a required residence time of >23 h.

## Conclusions

A VFR was shown to continuously remove up to 85% (average 67%) of the total Fe from AMD over a 10 month period with an equivalent footprint of 154 m^2^ per L/s of mine water flow, 0.61 m mean hydraulic head and residence times of >23 h. The VFR maintained a consistent flow throughout the trial. Chemical analysis as well as XRD suggest that the mineralogy of the VFR sludge is dominated by schwertmannite. The settling velocity of the VFR sludge was high for passive treatment sludge (3.4 m/h) and the sludge from the VFR visually resembled polymer-dosed sludge from active treatment systems. Analysis of the Cwm Rheidol influent has shown the prevalence of *‘Fv. myxofaciens*’, an EPS forming, Fe(II) oxidizing bacteria in the mine water. A centrifuge study indicated that the Fe-filt fraction has a size of below <35 nm. Iron removal mechanisms identified for the VFR system included the following: (i) filtration of particulate Fe(III) precipitates (>0.22 µm) by the accreting bed of precipitates; (ii) aggregation and filtration of nanoparticulate Fe(III) minerals (<0.22 µm) and/or heterogeneous precipitation of Fe(III) minerals; (iii) microbial Fe(II) oxidation and precipitation of Fe(III) minerals. This study represents an important contribution to understanding passive treatment of AMD and mechanisms of iron accretion from acidic waters. Ongoing studies at other AMD sites shall verify if the removal mechanisms described for the Cwm Rheidol VFR can be considered site specific or universal.
